# Antagonistic Bacterial Interactions Help Shape Host-Symbiont Dynamics within the Fungus-Growing Ant-Microbe Mutualism

**DOI:** 10.1371/journal.pone.0000960

**Published:** 2007-09-26

**Authors:** Michael Poulsen, Daniel P. Erhardt, Daniel J. Molinaro, Ting-Li Lin, Cameron R. Currie

**Affiliations:** 1 Department of Bacteriology, University of Wisconsin-Madison, Madison, Wisconsin, United States of America; 2 Department of Statistics, University of Wisconsin-Madison, Madison, Wisconsin, United States of America; University of Toronto, Canada

## Abstract

Conflict within mutually beneficial associations is predicted to destabilize relationships, and theoretical and empirical work exploring this has provided significant insight into the dynamics of cooperative interactions. Within mutualistic associations, the expression and regulation of conflict is likely more complex than in intraspecific cooperative relationship, because of the potential presence of: i) multiple genotypes of microbial species associated with individual hosts, ii) multiple species of symbiotic lineages forming cooperative partner pairings, and iii) additional symbiont lineages. Here we explore complexity of conflict expression within the ancient and coevolved mutualistic association between attine ants, their fungal cultivar, and actinomycetous bacteria (*Pseudonocardia*). Specifically, we examine conflict between the ants and their *Pseudonocardia* symbionts maintained to derive antibiotics against parasitic microfungi (*Escovopsis*) infecting the ants' fungus garden. Symbiont assays pairing isolates of *Pseudonocardia* spp. associated with fungus-growing ants spanning the phylogenetic diversity of the mutualism revealed that antagonism between strains is common. In contrast, antagonism was substantially less common between more closely related bacteria associated with *Acromyrmex* leaf-cutting ants. In both experiments, the observed variation in antagonism across pairings was primarily due to the inhibitory capabilities and susceptibility of individual strains, but also the phylogenetic relationships between the ant host of the symbionts, as well as the pair-wise genetic distances between strains. The presence of antagonism throughout the phylogenetic diversity of *Pseudonocardia* symbionts indicates that these reactions likely have shaped the symbiosis from its origin. Antagonism is expected to prevent novel strains from invading colonies, enforcing single-strain rearing within individual ant colonies. While this may align ant-actinomycete interests in the bipartite association, the presence of single strains of *Pseudonocardia* within colonies may not be in the best interest of the ants, because increasing the diversity of bacteria, and thereby antibiotic diversity, would help the ant-fungus mutualism deal with the specialized parasites.

## Introduction

Although mutually beneficial associations appear congenial, selection favoring individual partners using resources to facilitate their own selfish interests is predicted to destabilize cooperation [e.g. 1]; a paradox known as “the tragedy of the commons” [1]–[2]. This inherent conflict is recognized as shaping both within- and between-species cooperative relationships [3]–[6], and extensive theoretical and empirical studies examining conflict within intraspecific cooperative relationships have provided important insights into social evolution and the stability of cooperation [e.g.], [ 3], [ 7–8]. The main mechanisms enforcing cooperation involve the alignment of interests of partners [e.g.], [ kin selection theory: 7], [ 9–10] and sanctions against cheaters [e.g.], [worker policing, 3, 8].

The regulation of conflict may be of particular importance within mutualistic associations, where partners are unrelated so cooperation cannot be explained by kin selection [7]. Mechanisms to reduce conflict, analogous to those within intraspecific cooperative relationships, appear to also govern interspecific interactions [6], [11; for a recent review], [see 12]. Studies exploring conflict in mutualistic associations have documented alignment of interests through partner fidelity feedback, exemplified in joint mutualist transmission between host generations [vertical transmission; 13]–[16]. In addition, the presence of host sanctions directed towards cheating symbionts has been identified in several mutualisms [e.g.], [ 17]–[20].

Although studies exploring mutualisms in a pair-wise framework have provided important insights into mutually beneficial interactions, the costs and benefits of cooperation and cheating within mutualisms can be significantly altered by the interactions that take place within the more complex communities in which they occur [e.g.], [ 21–22]. For example, recent findings indicate that additional mutualistic and/or parasitic symbionts are often associated with mutualisms [e.g. 15], [ 23]–[26] and because these symbionts can alter the cost-benefit ratios within the focal bipartite association [27]–[30], they have the potential to shape the expression of conflict. Furthermore, populations of mutualists are composed of different symbiont genotypes, and conflict expression and regulation is likely to be influenced by host-mutualist genotype combinations as well as the avoidance of mixing of multiple genotypes within individual combinations [4]–[5], [31]–[32]. In addition to populations being composed of multiple genotypes of individual mutualistic species, a further level of complexity is the frequent presence of multiple species or strains of each mutualist lineage that can potentially form novel pairings of mutualists through host switches [cf. 16], [ 33]–[35]. Studies indicate that such novel pairings result in changes in the costs and benefits of the mutualism [e.g.], [ 36–37], and, thus, likely influence the expression and regulation of conflict between cooperative partners. Evaluating the compatibility among symbiont species as well as the costs and benefits of different mutualistic species combinations across host-symbiont coevolutionary histories may inform our understanding of the role of conflict in shaping evolutionary dynamics of mutualisms, including host specificity, coevolutionary dynamics, and the stability of mutualism.

This study evaluates the expression of conflict within a broader symbiotic community context, as well as across symbiont lineages, using the fungus-growing ant–microbe symbiosis, a model system in mutualistic cooperation. This system provides a relatively unique opportunity for studying the complexity of symbiont conflict expression, because: i) it is a complex community involving at least three obligately associated mutualists and one specialized parasite [15], [23]; ii) all four known symbionts are genetically and phylogenetically diverse, allowing for differences in cooperation and conflict between different genotype and species combinations to be investigated [34]–[36], [38]–[39]; iii) all three attine-associated symbiotic microbes can be cultured, allowing for interactions within and between symbiont lineages to be examined [15], [23], [32], [40]; iv) the coevolutionary histories of the symbiotic lineages have been explored [35], [ 41]–[42; Cafaro et al. in preparation]; and v) conflict is inherent and has been examined in the ant-cultivar association [32], [34], [36], [38]. Here we employ symbiont pairing assay experiments, designed with particular emphasis on the factors described above, to evaluate the expression of conflict in the ant-*Pseudonocardia* association.

### Cooperation and Conflict in the Fungus-Growing Ant–Microbe Symbiosis

Fungus-growing ants (Attini, Hymenoptera), a monophyletic group of more than 210 New World ant species, are characterized by their obligate dependence on fungi for food [41], [43]. The ants cultivate their mutualistic fungus by providing it substrate for growth and protection from unfavorable abiotic and biotic conditions [15], [23], [28], [44]–[47]. Several mechanisms are present within the ant-fungus association that help reduce conflict and enforce cooperation. Vertical (uniparental) transmission of the fungal mutualist from parent to offspring nests by female alates helps align the reproductive interests of the ants and their fungi [5]–[6], [13]–[14], [34]. Although, as predicted by this transmission mode, fungal symbiont lineages have evolved in parallel with lineages of their ant hosts on the broad phylogenetic scale [e.g., 41], symbiont switching disrupts the expected pattern of strict lower-level phylogenetic ant-fungus congruence [36], [42], [48]–[49]. The observation that host-symbiont switches are frequent, at least on an evolutionary time scale, indicates situations where the reproductive interests of the ants and fungi are decoupled and where mixing of genetically distinct fungus clones may incur competition [cf. 5]–[6; 32]. In the leaf-cutter ant *Acromyrmex*, competitive conflict between genetically distinct fungus clones is apparently prevented through behavioral recognition and removal of non-native fungi by the ants [36], [50]. Furthermore, the resident fungus imprints ant faecal droplets, used to fertilize new mycelial growth, and this imprinting elicits the removal of compounds and fragments of non-native fungi, so that the residing fungus enforce single-clone rearing by precluding the establishment of additional clones within colonies [32].

The ants and their fungal cultivar are only two members of a more complex symbiotic community. The fungal mutualist is parasitized by specialized and coevolved microfungi in the genus *Escovopsis* (Ascomycota: Hypocreales) [23], [35]. To help prevent and suppress *Escovopsis* infections the ants employ specific behaviors, including grooming their fungal mutualist and weeding out heavily infect garden fragments [47]. In addition, the ants engage in a second mutualism with actinomycetous bacteria (genus *Pseudonocardia*), which produce antibiotics capable of inhibiting the growth of the specialized parasite [15], [28], [45], [51]. The antibiotic-producing bacteria are typically housed in elaborate structures on the cuticle of workers, connected to exocrine glands that appear to provide nutrients to support the growth of *Pseudonocardia* [52]. The *Escovopsis* and *Pseudonocardia* symbionts are phylogenetically diverse and have an ancient and coevolved association with fungus-growing ants and their fungal cultivar [35], [ 39]–[40], [ 51]–[52; Cafaro et al. in preparation]. Individual ant colonies appear to associate with a single strain of the actinomycetous bacterium, but different ant species, and even genera, maintain very closely related *Pseudonocardia* symbionts [39]. Thus, even though the default vertical transmission mode predicts congruence between ant and bacterial phylogenies [15], [45], horizontal exchanges of *Pseudonocardia* occur over evolutionary time and disrupt strict cocladogenesis (Cafaro et al. in preparation).

Although the ant-actinomycete mutualism is expected to be stabilized by the alignment of interests of specific symbiont pairs through vertical transmission and the rearing of single strains of *Pseudonocardia* within individual ant colonies [5]–[6], [15], [39], potential ant-bacterium conflict can be envisioned. Horizontal transfer of *Pseudonocardia* decouples the reproductive interests of the ants and the bacteria, and can result in the presence of more than a single genotype of *Pseudonocardia* within individual nests [cf. 39]. Further conflict is envisioned because *Pseudonocardia* produces antibiotics to help defend the ants' fungus garden from the horizontally transmitted *Escovopsis* parasite [15], [23]. Observed variation in the inhibitory capabilities of different *Pseudonocardia* strains suggests that the actinomycetous symbionts are involved in Red-Queen-like antagonistic co-evolution with the parasite *Escovopsis* (Poulsen et al. in preparation), inferring that individual ant colonies would benefit from maintaining more than a single genotype of *Pseudonocardia* or from being able to easily acquire novel strains when confronted with resistant parasite strains. Replacement by, or mixing with, a foreign strain of *Pseudonocardia* would incur a direct cost to the resident *Pseudonocardia* strain, which would either be discarded or have to share both the energy allocated by the ants for growth and the transmission to the next generation on the cuticle of prospective ant queens [cf. 5]–[6]. A resident *Pseudonocardia* strain would therefore benefit from preventing foreign strains from invading its niche, and one likely mechanism to do so would be the production of compounds that inhibit other bacteria. Such antagonistic interactions are common in free-living bacteria [e.g.], [ 53–54] by means of the production of bacteriocidal compounds usually targeted at conspecifics [bacteriocins; for a review, see 55].

In this study we examine the presence, frequency, and intensity of antagonistic interactions between pairs of *Pseudonocardia* symbionts originating from different fungus-growing ant colonies. We explore this through Petri dish bioassays pairing symbionts spanning the phylogeny of *Pseudonocardia* associated with fungus-growing ants as well as free-living (non-ant associated) actinomycetes. In addition, we conduct a second set of bioassay challenges specifically focusing on a phylogenetically narrow group of strains isolated from sympatric and allopatric species of the leaf-cutting ant genus *Acromyrmex*. We evaluate the factors contributing to the observed patterns of antagonistic interactions, including the species or genus of ant host for the symbiotic *Pseudonocardia* strains. Additionally, we examine the relationship between antagonism and relative genetic difference between pairs of strains inferred from patterns of DNA sequence divergence analyses.

## Methods

### Experiments and bacterial isolates

We conducted two sets of bioassay experiments. The first examined interactions between strains of *Pseudonocardia* symbionts associated with fungus-growing ant species spanning most of the phylogenetic diversity of the ant-microbe mutualism (cross-phylogeny bioassay experiment). Fourteen *Pseudonocardia* isolates were obtained from eight species of fungus-growing ants, including: *Apterostigma dentigerum* (belonging to one of the most phylogenetically basal ant genera), *Cyphomyrmex costatus*, *C. muelleri*, *C. longiscapus* (*Cyphomyrmex* being a phylogenetically basal/intermediate ant genus), *Trachymyrmex zeteki, T. cornetzi* (*Trachymyrmex* being a phylogenetically intermediate to derived non-leaf-cutting ant genus), *Acromyrmex echinatior* and *A. octospinosus* (*Acromyrmex* being one of the most derived attine ant genera (a leaf-cutter) [56]. Four free-living actinomycetes obtained from the USDA ARS culture collection (http://nrrl.ncaur.usda.gov; see Table 1) (*Streptomyces griseus*, *Pseudonocardia saturnae*, *P. thermophilia*, and *P. halophobica*), and a *Nocardiodes* sp., culture isolated from a colony of *A. dentigerum,* were also included in this experiment. The second bioassay experiment examined possible antagonism between bacterial symbionts associated with sympatric and allopatric species of the leaf-cutting ant genus *Acromyrmex* (within-*Acromyrmex* bioassay experiment). Specifically, we used 12 *Pseudonocardia* symbionts obtained from ant colonies from two geographically distant locations: Panama and Argentina. Eight strains originated from two sympatric Panamanian ant species (*A. echinatior* and *A. octospinosus*) collected from 2001–2005, and four strains were isolated from three ant species collected in Argentina in 2003 (one isolate from *A. niger,* two isolates from *A. hispidus fallax*, and one isolate from *A. laticeps*). The species, colony code, and geographic origin for each ant colony from which *Pseudonocardia* symbionts were isolated are provided in Table 1.

**Table 1:** Ant-associated and free-living actinomycete isolates used for the cross-phylogeny and within-*Acromyrmex* bioassay experiments.

Ant species origin	Colony ID/NRRL number	Geographic origin
**Cross-phylogeny Petri dish bioassay**		
*Apterostigma dentigerum* (1)	AL040114-11	Panama
*Apterostigma dentigerum* (2)	MTP050505-10	Panama
*Apterostigma dentigerum* (3)	AL050512-17	Panama
*Cyphomyrmex muelleri* (4)	AL050512-19	Panama
*Cyphomyrmex longiscapus* (5)	ST040117-7	Panama
*Cyphomyrmex costatus* (6)	CC031210-9	Panama
*Trachymyrmex cornetzi* (7)	AL041002-3	Panama
*Trachymyrmex cornetzi* (8)	AL041005-10	Panama
*Trachymyrmex zeteki* (9)	AL030107-17	Panama
*Trachymyrmex zeteki* (10)	AL050513-4	Panama
*Acromyrmex octospinosus* (11)	UGM020518-5	Panama
*Acromyrmex octospinosus* (12)	CC031210-22	Panama
*Acromyrmex echinatior* (13)	CC031212-1	Panama
*Acromyrmex echinatior* (14)	CC031209-2	Panama
*Streptomyces griseus* (15)	NRRL B-2165	
*Nocardiodes* sp. (16)	*A.dentigerum* AL050511-10	Panama
*Pseudonocardia saturnae* (17)	NRRL B-16172	
*Pseudonocardia halophobica* (18)	NRRL B-16514	
*Pseudonocardia thermophilia* (19)	NRRL B-1978	
**Within-** ***Acromyrmex*** ** Petri dish bioassay**		
*Acromyrmex niger* (A)	CC030327-2	Argentina
*Acromyrmex hispidus fallax* (B)	SP030327-1	Argentina
*Acromyrmex hispidus fallax* (C)	UGM030327-2	Argentina
*Acromyrmex laticeps* (D)	UGM030330-4	Argentina
*Acromyrmex echinatior* (E)	291	Panama
*Acromyrmex echinatior* (F)	292	Panama
*Acromyrmex echinatior* (G)	295	Panama
*Acromyrmex echinatior* (H)	CC031212-1	Panama
*Acromyrmex octospinosus* (I)	CC011010-4	Panama
*Acromyrmex octospinosus* (J)	CC031210-22	Panama
*Acromyrmex octospinosus* (K)	ST040116-1	Panama
*Acromyrmex octospinosus* (L)	UGM020518-5	Panama

Colony IDs are given for isolates from fungus-growing ant colonies; NRRL numbers are given for the free-living actinomycetes obtained from the USDA ARS culture collection (http://nrrl.ncaur.usda.gov). Isolates are listed according to ant species origin, the strains involved in the cross-phylogeny bioassay are numbered 1–19, and the strains involved in the within-*Acromyrmex* bioassay are labeled A-L.

### Actinomycete symbiont isolations

The *Pseudonocardia* symbionts used in this study were obtained using a modified method from Cafaro and Currie [51]. Actinomycete isolations were predominantly done on ants tending the fungus garden, since these workers carry the highest biomass of *Pseudonocardia* [28], [57]. Since only one strain of bacterium is expected to be associated with a given ant colony [cf. 39], isolation attempts were done until a *Pseudonocardia* strain was obtained; this meant performing isolations on between one and ten ants per colony. In the ant genera *Apterostigma* and *Cyphomyrmex*, actinomycetes were isolated by placing individual ants, either whole or ground-up, in 750 µl autoclaved water in a 1.5 ml eppendorf tube. The tube was mixed for ∼30 seconds using a vortex, and 100–250 µl of the suspension was subsequently spread onto chitin-agar plates containing antifungals (nystatin 10,000 units/ml and cycloheximide 5% *w*/*v*). To obtain *Pseudonocardia* symbionts from ants in the genera *Trachymyrmex* and *Acromyrmex*, bacteria were either isolated following the protocol describe above, or by aseptically scraping bacterial cells from the propleural plates with a scalpel [cf. 15], [57]. After 2–3 weeks of growth at room temperature, *Pseudonocardia* colonies were transferred to Petri plates containing yeast malt extract agar (YMEA) with antifungals (concentrations as above) [cf. 51].

### Bioassay challenges

**Figure 1:**
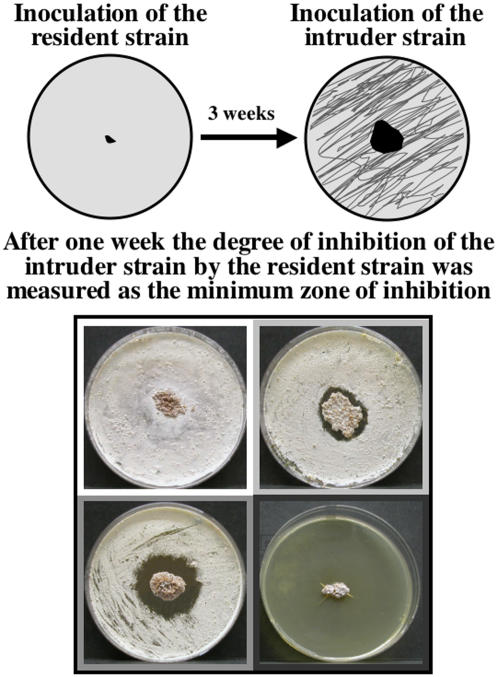
Schematic diagram of the assessment of actinomycete antagonism. The resident actinomycete was inoculated in the center of a Petri dish and left at room temperature for three weeks, after which a suspension of the intruder actinomycete was inoculated to the entire unoccupied Petri dish area (top). One week after the intruder strain was applied, the minimum diameter of the resident strain and the minimum zone of inhibition (zoi) imposed on the intruder strains were measured. The bottom section of the figure shows four examples of actinomycete-actinomycete reactions, with no antagonism displayed by the residing clone towards the intruding actinomycete (top left), intermediate levels of antagonism with slight to strong inhibition (top right and bottom left), and to complete inhibition of the intruder by the residing strain (bottom right). Pictures are framed with colors according to the size of the zoi: white = no inhibition, light grey = 0.01–0.29 cm, grey = 0.30–0.59 cm, darker grey = 0.60–0.89 cm, and darkest grey>0.90 cm.

In both experiments, biossay challenges involved co-culturing two strains of actinomycetes on YMEA with antifungals (concentrations as above) in 8.5 cm diameter Petri dishes. The first strain (hence forth referred to as the “resident” strain) was point inoculated in the center of the Petri dish and left to grow at room temperature for three weeks (Fig. 1). The second strain (hence forth referred to as the “intruder” strain) was inoculated to the entire unoccupied Petri dish area by applying 200 µl of autoclaved water containing a suspension of approximately 800–1200 bacterial cells per µl (Fig. 1). The plates were checked regularly until the intruder strain had sufficient time to grow to fill the Petri plate or a distinct zone of inhibition (zoi) imposed by the resident strain had established and no longer changed over time. When present, typically one week after the intruder strain was applied, the minimum zone of inhibition (zoi) imposed on the intruder strains were measured as well as the minimum diameter of the resident strain (Fig. 1). Actinomycete strains were paired in all possible combinations, 361 in the cross-phylogeny experiment and 144 in the within-*Acromyrmex* experiment, and three replicates were performed for each pairing. In the cross-phylogeny bioassay experiment the three replicates for each pairing were applied on three succeeding days, because the application of the second strain was labor intensive.

### Genetic comparison of actinomycete strains

To infer phylogenetic relationships between *Pseudonocardia* strains used in the experiments, and to determine if genetic distance between strains influences antagonistic reactions, we did partial sequencing of ribosomal 16S rDNA and nuclear Elongation Factor-Tu (EF-Tu) regions using standard PCR and sequencing techniques [39], [51], [58]. PCR amplicons, generated using already published primers [39], [51], were sequenced at the UW-Madison Biotechnology Center (http://www.biotech.wisc.edu). The resulting sequences were corrected for mismatches using Sequencher 4.6 for Windows (Gene Codes Corporation, Ann Arbor, MI), and aligned using Clustal X 1.83 [59] and MacClade 4.07 OS X [60]. Phylogenetic analyses were performed in PAUP* 4.0b 10 [61] and bootstrap support was assessed with 1000 pseudoreplicates under Maximum Parsimony (MP), Maximum Likelihood (ML), and Neighbor-Joining (NJ) conditions. Prior to performing the ML analyses, the models of sequence evolution were determined in Modeltest 3.7 [62]. Gaps in the sequences were treated as missing data. For the strains used in the cross-phylogeny bioassay, the model was General Time Reversible (GTR) with among-site substitution rate variation (R[A-C] = 0.6331, R[A-G] = 1.3004, R[A-T] = 0.8053, R[C-G] = 1.6249, R[C-T] = 2.4121, and R[G-T] = 1.0000), the proportion of invariable sites (I) being 0.4427, and a gamma distribution shape parameter (G) of 0.6005. The same model of sequence evolution governed the sequences of strains included in the within-*Acromyrmex* bioassay, with R[A-C] = 0.5817, R[A-G] = 0.7977, R[A-T] = 0.5239, R[C-G] = 1.0821, R[C-T] = 1.8745, and R[G-T] = 1.0000, I = 0.7055, and G = 0.6718. Finally, the frequency of base pair differences between pairs of actinomycete strains (relative genetic distance) was determined in PAUP* 4.0b 10 [61] and inserted as a continuous covariate in the statistical analyses (Tables 2 and 3; see below). All sequences are available from GenBank (accession numbers EF588206, EF588217-8, EF588223-5 EF588228-9, EF588231, EF588235, EF588246-7, EF588252-4, EF588257-8, EF588260, EU139566-603).

**Table 2:** Relative genetic distances between pairs of actinomycetes involved in the across-phylogeny bioassay

	1	2	3	4	5	6	7	8	9	10	11	12	13	14	15	16	17	18	19
**1**	0.000																		
**2**	0.004	0.000																	
**3**	0.004	0.002	0.000																
**4**	0.079	0.075	0.073	0.000															
**5**	0.074	0.073	0.071	0.009	0.000														
**6**	0.077	0.073	0.071	0.003	0.009	0.000													
**7**	0.033	0.033	0.034	0.067	0.063	0.066	0.000												
**8**	0.006	0.006	0.006	0.079	0.077	0.077	0.036	0.000											
**9**	0.033	0.032	0.031	0.065	0.061	0.064	0.006	0.035	0.000										
**10**	0.076	0.079	0.080	0.082	0.081	0.081	0.069	0.081	0.068	0.000									
**11**	0.003	0.003	0.002	0.073	0.071	0.071	0.033	0.004	0.031	0.079	0.000								
**12**	0.035	0.035	0.033	0.066	0.062	0.065	0.005	0.037	0.007	0.072	0.035	0.000							
**13**	0.032	0.036	0.036	0.068	0.064	0.068	0.009	0.036	0.009	0.073	0.033	0.009	0.000						
**14**	0.034	0.038	0.038	0.070	0.066	0.069	0.010	0.037	0.011	0.074	0.034	0.011	0.005	0.000					
**15**	0.119	0.118	0.118	0.114	0.114	0.114	0.114	0.121	0.114	0.120	0.122	0.118	0.123	0.122	0.000				
**16**	0.072	0.078	0.077	0.110	0.109	0.111	0.082	0.073	0.084	0.120	0.080	0.083	0.084	0.083	0.124	0.000			
**17**	0.065	0.062	0.062	0.079	0.075	0.077	0.055	0.064	0.056	0.098	0.058	0.058	0.058	0.058	0.131	0.111	0.000		
**18**	0.067	0.065	0.063	0.059	0.061	0.057	0.061	0.067	0.058	0.070	0.066	0.062	0.066	0.062	0.125	0.110	0.069	0.069	
**19**	0.081	0.084	0.083	0.064	0.066	0.063	0.082	0.086	0.081	0.088	0.080	0.084	0.080	0.081	0.121	0.122	0.080	0.000	0.000

The frequency of base pair differences (relative genetic distance), averaged over partial sequences of the 16S and EF-Tu regions (1393 bp and 1004 bp, respectively), between actinomycete strains involved in the cross-phylogeny bioassay. Averaged, Isolates are listed according to ant species origin and the strains are numbered 1–19 (see Table 1).

**Table 3:** Relative genetic distances between pairs of actinomycetes involved in the within-*Acromyrmex* bioassay

	A	B	C	D	E	F	G	H	I	J	K	L
A	0.000											
B	0.035	0.000										
C	0.032	0.008	0.000									
D	0.033	0.012	0.010	0.000								
E	0.033	0.003	0.006	0.011	0.000							
F	0.030	0.008	0.007	0.011	0.007	0.000						
G	0.034	0.003	0.006	0.010	0.001	0.006	0.000					
H	0.033	0.009	0.009	0.007	0.008	0.012	0.007	0.000				
I	0.040	0.004	0.004	0.005	0.002	0.002	0.002	0.006	0.000			
J	0.033	0.009	0.009	0.007	0.007	0.012	0.008	0.004	0.008	0.000		
K	0.031	0.013	0.012	0.006	0.012	0.007	0.011	0.008	0.004	0.009	0.000	
L	0.004	0.036	0.034	0.036	0.035	0.030	0.036	0.032	0.040	0.033	0.033	0.000

The frequency of base pair differences (pair-wise relative genetic distances), averaged over partial sequences of the 16S and EF-Tu regions (1394bp and 959bp, respectively), between actinomycete strains involved in the within-*Acromyrmex* bioassay. Colonies are labeled A-L according to Table 1.

### Statistical analyses

Due to the non-normality of the data, and the high sensitivity of 0-values in log-linear models, the statistical approach used to analyzing the bioassays was a generalized linear model design with an underlying gamma distribution. The exact values of the zoi measurements were inserted in the analyses, except that 0-values were imputed with 0.0001. All analyses were performed using SAS/STAT software, Version 9.1.3 of the SAS System for Microsoft Windows (Copyright ©2007 SAS Institute Inc. SAS and all other SAS Institute Inc. product or service names are registered trademarks of SAS Institute Inc., Cary, NC, USA), and significance of terms in the models was determined through Likelihood Ratio Statistics for Type III ANOVAs.

Two models were built for each of the bioassay experiments. A nested model contained ant host of resident and intruder actinomycetes (genus in the cross-phylogeny bioassay, species in the within-*Acromyrmex* bioassay), resident and intruder actinomycete strains nested within their respective ant origins, and the interaction between ant hosts of the two strains. The minimum diameter of the resident actinomycete at the time the zoi of the intruder actinomycete was scored, and the relative genetic distance between pairs of actinomycetes were inserted as continuous covariates. Due to the nested design of the models, they could not include the interaction term between the resident and the intruder actinomycete strains. An additional model was therefore built for each bioassay, including only resident actinomycete strain, intruder actinomycete strain, the resident*intruder interaction term, and the size of the resident actinomycete inserted as a continuous covariate.

Finally, in order to test if the same level of inhibition was obtained when switching the order of inoculation of a given pair of strains (i.e. if reactions displayed were symmetrical), Mantel tests of matrix associations with 10,000 permutations were performed on reactions displayed in both bioassays using Arlequin 2.0 [63].

## Results

**Figure 2:**
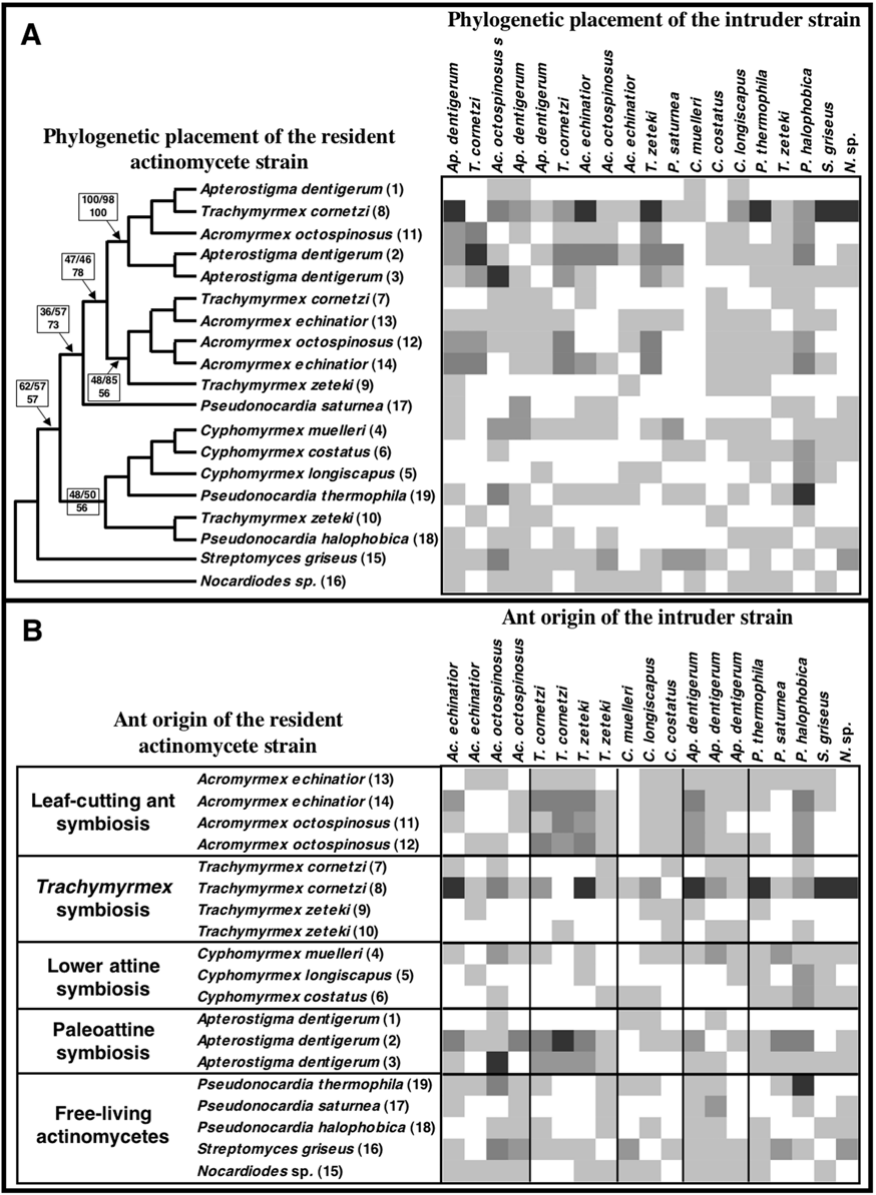
Antagonistic reactions across the phylogeny of *Pseudonocardia* associated with attine ants. Average degree of antagonism from the resident towards the intruder actinomycete for the 361 combinations of actinomycetes, spanning the attine phylogeny and including five free-living actinomycetes. Ant-associated *Pseudonocardia* strains are labeled with the genus and species name of the ants they were isolated from, and all strains are numbered as in Table 1 to distinguish strains originating from the same ant species. Different shades of grey denote strength of inhibition (average size of zone of inhibition, zoi; n = 3): white = no inhibition, light grey = 0.01–0.29cm, grey = 0.30–0.59cm, darker grey = 0.60–0.89cm, and darkest grey = above 0.90cm. A) Shows the bioassay results organized according to the phylogenetic placement of the nineteen strains paired. The phylogeny is based on 1393bp of 16S and 1004bp of EF-Tu. Bootstrap support values after 1000 pseudoreplicates under MP (top, left), ML (top, right), and NJ (bottom) conditions are given for the branches separating the major clades in the phylogeny (see text for details). B) Shows the same bioassay result organized according to the ant origin of the actinomycetes: leaf-cutting ants, *Trachymyrmex* ants, the lower attines, paleoattines, or free-living.

Our cross-phylogeny bioassay experiment, pairing strains of *Pseudonocardia* isolated from different colonies of attine ants spanning most of the diversity of the tribe and some non-ant associated free-living isolates, revealed a high proportion (208 out of 342) of pairings of actinomycetes with detectable levels of inhibition of the intruder strain by the resident strain (Fig. 2). Although antagonism was common, the ability to inhibit other bacteria varied significantly, with individual strains inhibiting between four (21.1%) and 17 (89.5%) of the intruder strains (on average 57.9%, 11 of 19; a significant effect of the resident strain origin in the nested model: Table 4, p<0.0001) (Fig. 2). As expected from the normal synchronization of the expression of genes coding for secondary metabolites and resistance in bacteria [e.g., 64], all *Pseudonocardia* strains were resistant to their own compounds (diagonal in 1+Fig. 2). While all 19 intruder strains were susceptible to inhibition by at least six resident strains, they all exhibited resistance to compounds produced by some strains (ranging from 3–12 of pairings of actinomycetes from different colonies, average seven), resulting in a significant effect of the intruder strain (Table 4, nested model: p<0.0001) (Fig. 2). Thus, the outcome of a given pair-wise interaction was influenced by the combination of the strength of inhibition by the resident strain and the degree of intruder susceptibility (Table 4, statistical interaction term in actinomycete origin model: p<0.0001). The Mantel test of matrix correlation indicated asymmetry in pairs of strains; the same level of inhibition was not necessarily obtained when switching the order of inoculation of a given pair of strains (Mantel r = −0.09; p = 0.8562). Symbiont strains isolated from the same ant genus displayed more similar inhibition and susceptibility profiles compared to strains isolated from different ant genera (Table 4, nested model: p<0.0001 and p = 0.005 for resident and intruder strain ant genus origin, respectively; Fig. 2B). The relative genetic distance between pairs of actinomycetes also had a significant effect on inhibition (Table 4, nested model: p = 0.0078), suggesting that antagonism is more common between more distantly related pairs of strains. The size of the resident strain did not affect the degree of antagonism (Table 4, nested model: p = 0.6892).

**Table 4:** Statistical results of the generalized linear models with underlying gamma distributions for the two Petri dish bioassays.

**Cross-phylogeny Petri dish bioassay**		
	Nested model	Model with only strain origin
Resident	χ^2^ = 266.6, df = 14, p<0.0001	χ^2^ = 1339.9, df = 18, p<0.0001
Intruder	χ^2^ = 88.44, df = 14, p<0.0001	χ^2^ = 631.67, df = 18, p<0.0001
Ant genus of resident	χ^2^ = 40.83, df = 4, p<0.0001	
Ant genus of intruder	χ^2^ = 14.86, df = 4, p = 0.0050	
Size of resident	χ^2^ = 0.160, df = 1, p = 0.6892	χ^2 = ^0.040, df = 1, p = 0.8396
Relative genetic distance	χ^2^ = 7.08, df = 1, p = 0.0078	
Resident*Intruder		χ^2 = ^2099.2, df = 324, p<0.0001
Ant genus of resident * Ant genus of intruder	χ^2^ = 84.67, df = 16, p<0.0001	
**Within-** ***Acromyrmex*** ** Petri dish bioassay**		
	Nested model	Model with only strain origin
Resident	χ^2^ = 123.7, df = 7, p<0.0001	χ^2^ = 943.5, df = 11, p<0.0001
Intruder	χ^2^ = 248.8, df = 7, p<0.0001	χ^2^ = 1003.1, df = 11, p<0.0001
Ant species of resident	χ^2^ = 110.2, df = 4, p<0.0001	
Ant species of intruder	χ^2^ = 37.8, df = 4, p<0.0001	
Size of resident	χ^2^ = 6.85, df = 1, p = 0.0089	χ^2^ = 0.51, df = 1, p = 0.4735
Relative genetic distance	χ^2^ = 11.62, df = 1, p = 0.0007	
Resident*Intruder		χ^2^ = 1307.6, df = 121, p<0.0001
Ant species of resident * Ant species of intruder	χ^2^ = 73.51, df = 16, p<0.0001	

The top section gives the results for the cross-phylogeny bioassay and the bottom section gives the results for the within-*Acromyrmex* bioassay. For both bioassays, a nested model and a model including only resident, intruder, and their interaction were built (see text for detail). Significance of terms in the models was determined through Likelihood Ratio Statistics for Type III ANOVAs, χ^2^-values, degrees of freedom (df), and p-values are given.

**Figure 3:**
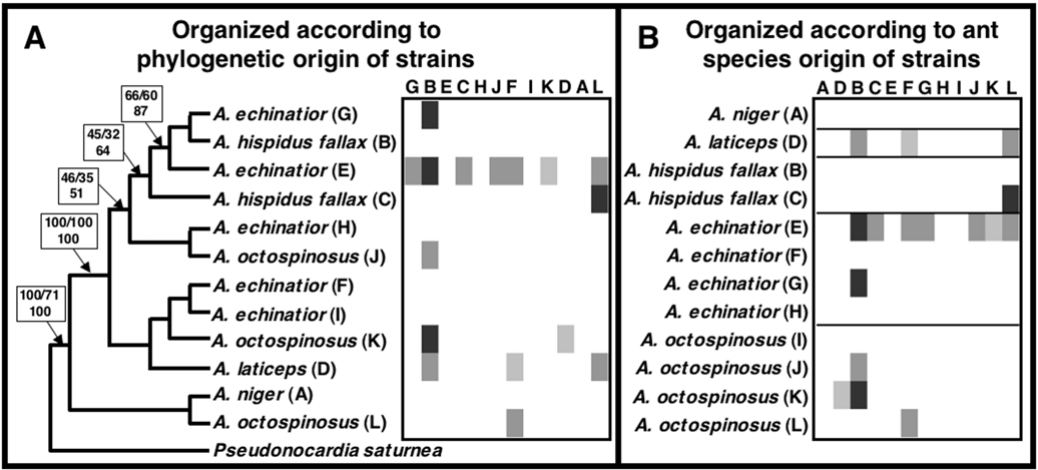
Antagonistic reactions between *Pseudonocardia* strains associated with *Acromyrmex* leaf-cutting ants. Average degree of antagonism from the resident towards the intruder actinomycetes for the total of the 144 combinations performed within and between actinomycete strains isolated from five species of *Acromyrmex*. Strains are labeled after the ant species they were isolated from, and strains from the same ant species are distinguished by their labeling (A through L). Different shades of grey denote strength of inhibition (average size of zone of inhibition, zoi; n = 3): white = no inhibition, light grey = 0.01–0.29cm, grey = 0.30–0.59cm, darker grey = 0.60–0.89cm, and darkest grey = above 0.90cm. A) Shows the bioassay results organized according to the phylogenetic placement of the twelve strains paired, based on 1394bp of 16S and 959bp of EF-Tu sequences; bootstrap support values after 1000 pseudoreplicates under MP (top, left), ML (top, right), and NJ (bottom) conditions are given for the branches separating the major clades. B) Shows the bioassay results organized according to the ant species origin of the actinomycetes, with horizontal lines separating reactions displayed by isolates from the five ant species.

In the within-*Acromyrmex* bioassay experiment, antagonism was uncommon, with only 16 of the 132 pairings of actinomycetes originating from different colonies (12.1%) exhibiting inhibition (Fig. 3). There was a significant difference in the ability of *Acromyrmex* symbionts to inhibit other strains, with five strains showing no sign of antagonism, four strains inhibiting just a single intruder, and one strain inhibiting seven of the intruders (strains inhibited on average 1.3 intruders; Table 4, nested model: p<0.0001). There was also a significant difference in the strains resistance to inhibition: four of the intruders were not susceptible to inhibition by any of the resident strains and the remaining strains were inhibited by 1–5 strain(s) (on average two; Table 4, nested model: p<0.0001). Similar to the cross-phylogeny assay, the interaction between resident and intruder actinomycetes was even stronger than the isolated factors alone (Table 4, actinomycete origin model: p<0.0001). Furthermore, the degree of antagonism was affected by the host ant species for both the resident (p<0.0001) and intruder (p<0.0001) symbionts (Table 4, nested model; Fig. 3B). In addition, we found a significant effect of the relative genetic distance between pairs of strains, indicating a genetic effect on the degree of antagonism (Table 4, nested model: p = 0.0007; Fig. 3A). Finally, contrasting the cross-phylogeny bioassay, the size of the resident strain had a significant effect on the observed antagonism (Table 4, nested model: p = 0.0089). However, size of strain was no longer significant if the host ant species for the symbionts were not included in the model (Table 4, actinomycete origin model: p = 0.4735). As in the cross-phylogeny bioassay, the observed reactions were not symmetric (Mantel r = −0.07; p = 1.00), so that reactions were dependent upon the order of strains used in a given pairing.

## Discussion

Our bioassay experiments revealed the presence of antagonistic interactions among *Pseudonocardia* symbionts of fungus-growing ants. Conflict reactions between strains were observed in more than half the pairings of actinomycetes isolated from across the fungus-growing ant tribe, with most strains being capable of inhibit the growth of at least 20% of the actinomycetes they were tested against. The widespread ability of ant-associated *Pseudonocardia* to inhibit other symbiont strains suggest that competitive conflict will often be expressed when actinomycete strains mix and thereby may directly impact host-symbiont dynamics. Furthermore, the presence of reactions across the coevolutionary history of the ant-actinomycete association suggests that bacteria-bacteria antagonism has been present since the origin of the fungus-growing ant–microbe symbiosis.

Although our results indicate that *Pseudonocardia* bacteria have the capacity to inhibit other *Pseudonocardia* strains, there was abundant variation in both the ability to inhibit other strains and in the resistance to compounds. Inhibition is more likely between pairs of symbionts associated with more distantly related genera of fungus-growing ants, since antagonism was far more common in our cross-phylogeny experiment (57.9% of pairings revealed antagonism) as compared to pairings in the within-*Acromyrmex* bioassay experiment (12.1% of pairings displayed antagonism), where both the number and strength of antagonistic interactions were substantially lower. This is further supported by the level of antagonism in the cross-phylogeny experiment being significantly affected by the host ant genus origin of the bacterial symbionts. However, this effect may be at least partially due to the significant effect of genetic distance between pairs of strains (Figs. 2A, 3A), because individual ant species or genera rear relatively closely related strains compared to the overall diversity of *Pseudonocardia* associated with fungus-growing ants. The mixing of more distantly related actinomycete strains, often associated with different species or genera of ants, could thus incur stronger conflict expression when mixes occur between strains across the broad attine ant groups than within these groups, potentially impacting dynamics of host-symbiont switching (see below).

Although host ant genus and genetic distance between strains had a significant effect on antagonism observed between strains, they did not explain all the variation observed in our experiment. Across the diversity of *Pseudonocardia* symbionts, we found some strains that were highly potent (i.e., had the ability to inhibit a high proportion of the symbionts they were challenged with), as well as particularly resistant strains (i.e., with the ability to resist inhibition by a large proportion of resident strains). In both experiments the primary factors contributing to the variation in antagonism were i) the strength of inhibition by the residing bacterium, ii) the degree of susceptibility of the intruder, and iii) the combination of inhibitory capabilities and susceptibility (Figs. 2, 3; Table 4). This indicates that the outcome of interactions is strongly affected by the secreted secondary metabolites and the presence/absence of resistance to these secretions. This implies that strains with higher potency would be predicted to benefit from being able to resist replacement by a larger collection of actinomycetes, while strains able to resist inhibition would be predicted to be better at invading new colonies. Future work examining the costs associated with these phenotypic properties of different *Pseudonocardia* strains would provide important insights into the ant-bacteria mutualism.

The observed antagonistic reactions resemble interactions in free-living actinomycetes [53]–[54; this study], where they are believed to contribute significantly to microbial fitness in competition for nutrient and space resources [for a review, see 55]. Species of bacteria from all major lineages are known to secrete tens to hundreds of different biologically active proteins with a bacteriocidal mode of action (bacteriocins), which are believe to target mostly conspecifics [65]–[66 and references therein]. The observed antagonism between *Pseudonocardia* symbionts in this study is likely due to bacteriocins secreted to the surrounding medium. We suggest that selection on preventing the invasion and establishment of competing strains has resulted in the capacity of *Pseudonocardia* symbionts to uphold production of these compounds. Although it is possible that the observed antagonism is a result of the antibiotics produced by *Pseudonocardia* to inhibit the garden pathogen *Escovopsis*, this appears to be unlikely for several reasons. First, the antibiotics produced target the *Escovopsis* fungal parasites, and are thus expected to target the physiology of eukaryotes and not prokaryotes. Second, the pathogenic specificity of *Escovopsis* towards the ants' cultivar [35], [40], [67]–[68] and the variability in potency of the *Pseudonocardia*-derived antibiotics against *Escovopsis* (Poulsen et al. in preparation) suggest that actinomycete-*Escovopsis* interactions are governed by dynamics of antagonistic coevolution. This predicts that antibiotics targeted at *Escovopsis* are subject to specialization over evolutionary time and that inhibition of other microorganisms in the association, such as the mutualistic fungus and the actinomycete bacteria, should be diminished. Third, in a separate study, some of the same *Pseudonocardia* strains were used in symbionts-pairing assays with *Escovopsis* to examine the specificity of activity of antifungal against diverse strains of the parasite (Poulsen et al. in preparation). When comparing individual strains in these two studies, there was no correspondence in the inhibition profiles against other strains of *Pseudonocardia* and *Escovopsis* (i.e., *Pseudonocardia* strains that inhibited a similar collection of other actinomycete symbionts in this study, did not correspondingly inhibit a similar collection of *Escovopsis*). Since the chemical composition of secretions from *Pseudonocardia* remains unknown, it is not currently possible to determine whether bacteriocins or antifungals are responsible for the actinomycete-actinomycete antagonism. However, even if antagonism is a bi-product of antibiotics targeting *Escovopsis*, activity against other strains of *Pseudonocardia* would still shape the dynamics of the association by precluding the rearing of bacterial strains exhibiting antagonistic interactions within individual ant colony.

The presence of antagonism infers that competitive conflict may occur between pairs of actinomycetes if multiple genotypes of actinomycete symbionts are present within an individual colony. Mixing likely occurs during events of horizontal transfers of *Pseudonocardia*, known to take place on both an ecological and an evolutionary time scale [39; Cafaro et al. in preparation]. As discussed above, the effect of ant host genus in the cross-phylogeny experiment, as well as the antagonistic interactions being significantly less common in the within-*Acromyrmex* experiment, supports relatively more potent reactions between actinomycetes originating from different host ant genera. This indicates that the potential for conflict is likely less pronounced if mixes occur between more closely related ants, while stronger reactions between actinomycetes reared by different ant genera could restrict switching of bacterial lineages across the attine tribe, contributing to the maintenance of host-mutualist cocladogenesis (Fig. 2A; Cafaro et al. in preparation). While it is not in the interest of the actinomycetes to share ant-allocated resources, the display of antagonism indicates that events of mixing may incur a significant allocation of resources to competition rather than actinomycete growth and thereby also a potential reduction in the benefits of the association to the ants [cf. 5]–[6]. All available evidence indicates that individual colonies rear only a single actinomycete strain [39; Cafaro et al. in preparation], and considering the bipartite ant-actinomycete association (absence of parasitism), single-strain rearing would be in the interest of both ants and *Pseudonocardia* [cf. 5]–[6], [39]. Behavioral tests support that *Acromyrmex* ants preferentially choose their resident actinomycete over very closely related non-native strains, indicating the presence of a mechanism that further facilitates avoidance of multiple competing actinomycete strains within colonies [58].

In the respective bipartite associations between attine ants and their fungal and bacterial mutualists, default vertical symbiont transmission aligns the reproductive interests of the partners [13]–[15] and single symbiont rearing within individual colonies prevents the expression of competitive symbiont-symbiont conflict [32], [39]. Both ant-mutualist associations occasionally experience horizontal transmission, potentially decoupling the reproductive interests of host and symbionts and incurring competition between mixed symbiont strains. Furthermore, both symbionts have the ability to express antagonism that facilitates the maintenance of single symbionts within individual colonies. However, in the host-pathogen association between ants, cultivar, and actinomycete on one side and *Escovopsis* on the other, partner interests likely change. The rearing of a single fungal clone within a colony is not expected to pose a cost to the ants in relation to increased colony susceptibility to infection, because the cultivar itself does not have an efficient defense against typically infecting *Escovopsis* [68; Poulsen et al. in preparation; Little et al. in preparation]. However, the rearing of a single strain of actinomycetes could constrain the effectiveness of the ant-*Pseudonocardia* symbiosis, because multiple strains of antibiotic-producing bacteria within individual colonies would increase the diversity of compounds produced to defend against *Escovopsis* (Poulsen et al. in preparation). While single-strain rearing within individual colonies nevertheless is apparent, the extent to which horizontal switches of the bacterial mutualists take place appears to be symptomatic of the role they play in the association. Even though ant-actinomycete phylogenetic comparisons reveal some degree of host-mutualist cocladogenesis, actinomycete switches appear to take place more frequently than ant-fungus exchanges, including across the broad groupings governing ant-fungus associations (Fig. 2A; Cafaro et al. in preparation). This suggests that while ant recognition of actinomycete symbionts may aid in the prevention of conflict between different strains, it may at the same time allow for the ants to actively pursue replacing their resident actinomycete strain to acquire novel antibiotics against *Escovopsis*.

Conflict is an inherent major force shaping mutualistic interactions [4]–[6]. Studies exploring conflict within the fungus-growing ant–microbe symbiosis have identified the presence of behavioral ant-fungus incompatibilities and direct antagonism between fungal symbionts associated with different colonies, indicating that conflict shapes the mutualistic association [32], [34], [36], [38]. Our findings indicate additional layers of complexity of conflict expression within the fungus-growing ant microbe–symbiosis. This complexity is brought about through the widespread presence of antagonism between *Pseudonocardia* bacteria maintained by different ant colonies, species, and genera, but also by the presence of additional symbiont lineages (the *Escovopsis* parasites) that likely adjust the dynamics of cooperation and conflict within associations previously examined in a bipartite framework. Future studies exploring ant-bacteria and bacteria-cultivar conflict will inevitably provide additional insights into the expression and regulation of conflict within complex mutualistic associations.
